# Suppurative Keratitis

**Published:** 1892-02-27

**Authors:** 


### OPHTHALMOLOGY.

### SUPPURATIVE KERATITIS.

Suppuration of the cornea often arises quite spontaneously in feeble and aged persons, and is often provoked by the irritation caused by a foreign body under the lid. The strumous constitution is a predisposing cause, and a large percentage of cases occur in persons of the strumous diathesis; in such people the cornea, having practically no direct blood supply, is one of the weakest points in the body, and one of the first parts to suffer from inanition. The blood capillaries only extend about 1·5 mm. into the corneal substance, around its margin; it is therefore largely dependent on the surrounding network of capillaries for nourishment; and it is evident how easily any interference with the general blood supply will cause necrosis of its tissue.

It is often a complication of purulent conjunctivitis or traumatic iritis, and irido-cyclitis following the operation for extraction of cataract. The suppurative process may be diffuse or circumscribed. In the diffuse form the cornea loses its bright appearance, and looks like that of a dead fish, a grey cloudiness next coming over it, and gradually becoming a dirty yellow when the pus cells have invaded the layers of the cornea; destruction of the epithelium and the superficial layers of the cornea takes place, and the weakened tissues yield to the pressure of the intra-ocular fluids and bulge forward, forming anterior staphyloma, or goes on to rupture, leaving an unhealthy perforation in which the iris may become entangled, and which, in the event of healing, leaves the eye in an useless state, and sometimes even without perception of sight.

The circumscribed form of suppurative keratitis may attack any portion of the cornea, which portion will become dull; then an opaque spot is noticed with greyish surroundings, which become yellow when the pus has formed, and constitutes an abscess of the cornea. This, like abscesses elsewhere, will point, and if it point outwards forms an ulcer of the cornea, discharging its pus outwards; if inwards, the pus is discharged into the anterior chamber, and forms "hypopion." Sometimes it bursts both inwards and outwards, leaving a fistula.

If the abscess be at the lower part of the cornea the pus may gravitate between the layers and resemble an hypopion, from which it is distinguished by not changing its position with the patient's position, while the hypopion will, unless the discharge be very tenacious. The upper border of an hypopion is straight, that of the other condition may be curved or irregular, as the pus is confined between the layers of cornea; it is compared in appearance to the onyx, and called after it, "onyx of the cornea." It may, by perforating backwards and discharging its pus into the anterior chamber, be converted into an hypopion.

As regards treatment in suppurative keratitis, while all are agreed that everything to improve the patient's state of health and surroundings is necessary, by good food, stimulants, tonics, and good air, opinions are somewhat conflicting as regards the local treatment, more especially as regards the use of eserine and atropine. Some hold that the imminent danger of iritis calls imperatively for the use of atropine. Others consider that the use of eserine checks the migration of white corpuscles and limits the amount of suppuration, and is thus of primary importance. Fomentations to the eyelids in the early stages diminish pain and congestion, and light must be excluded as photophobia is a prominent symptom. Antiseptic lotions, among which quinine drops are in high repute, should be diligently used. When hypopion forms, the pus should be evacuated by paracentesis of the anterior chamber, or by Saemisch's operation, both of which operations have been previously described in these columns, so we will not repeat the methods of these procedures; but we would like to mention an operation recommended by Mr. Richard Williams, in which the pus is evacuated from the anterior chamber through a corneal incision, made through sound tissue, the knife being introduced into the anterior chamber from the lower border of the cornea, with its point directed to the centre of the pupil. It is, however, not allowed to come within the pupillary area, but it cuts out through the cornea again, short of that; through this incision the pus escapes readily, and he claims for it that: 1. It interferes less with the blood supply of the cornea. 2. The wound is in sound tissue, and therefore heals more readily. 3. There is less danger of prolapse of the iris.

Altogether it seems to have great advantages over the ordinary incision. These cases should be operated on before bulging has taken place, as ever so small an amount of bulging will cause interference with the vision. Iridectomy is sometimes called for in the early stages; the incision relieves the tension, and the portion of iris removed forms an artificial pupil, when the suppuration leaves an opacity of the cornea opposite the original pupil.

These cases are always ones accompanied with anxiety, and call for much skill on the part of the surgeon as to when and how to operate, for too long delay in deciding may be fatal to the eye.

